# Effects of Pueraria extracts on growth performance, immune function, and immune-related gene expression of Wuzhishan piglets

**DOI:** 10.3389/fvets.2025.1491130

**Published:** 2025-05-30

**Authors:** Hongzhi Wu, Weiqi Peng, Xilong Yu, Xiaoyu Zhang, Fengjie Ji, Qian Shen, Renlong Lv

**Affiliations:** ^1^Tropical Crops Genetic Resources Research Institute, Chinese Academy of Tropical Agricultural Sciences, Haikou, China; ^2^College of Animal Science and Technology, Northeast Agricultural University, Harbin, China; ^3^College of Animal Science and Technology, Henan University of Science and Technology, Luoyang, China; ^4^Hainan Xuhuai Technology Co. Ltd., Haikou, China

**Keywords:** Pueraria extracts, Wuzhishan piglets, growth performance, immune function, immune-related gene expression

## Abstract

Pueraria extracts contain phytoestrogens, particularly isoflavones such as daidzein and genistein, which have been used in traditional Chinese medicine for centuries due to their various health benefits. Wuzhishan pig is a small pig unique to China, native to Wuzhishan, Hainan Province. Pueraria extracts have been widely utilized in the production of various animals. However, there are relatively few reports regarding their application in piglets, particularly in local pig breeds. The experiment aimed to investigate the effects of dietary Pueraria extracts on the growth performance, immune function, and immune-related gene expression of Wuzhishan piglets. Forty-eight piglets were randomly divided into four groups, each with six replicates and two piglets in each replicate. The control group (CON) was fed the basal diet, and the other groups were given 400, 600, and 800 mg/kg Pueraria extracts (PE1, PE2, and PE3, respectively). Compared with the CON, (1) the dietary Pueraria extracts increased the average daily gain and decreased the feed conversion ratio in PE2 and PE3 (*p* < 0.05); (2) the dietary Pueraria extracts increased the serum immunoglobulin M and complement 3 and 4 contents in PE2 (*p* < 0.05); (3) the serum ALB and ALP contents in groups treated with the Pueraria extracts were higher (*p* < 0.05); (4) the ALT and GOT contents were lower (*p* < 0.05) in groups treated with Pueraria extracts; (5) the dietary Pueraria extracts increased the Bacillus acidi lactici and Saccharomycetes contents, and decreased the *Escherichia coli* and Salmonella contents in the jejunum in PE1, PE2, and PE3 (*p* < 0.05); (4) the dietary Pueraria extracts ameliorated the relative expression of *Interleukin-10*, *Tumor necrosis factor-α*, *Mucoprotein 2*, *Transforming growth factor-β1*, *Zonula occludens-1* in jejunum mucosa (*p* < 0.05). In this study, adding 600 mg/kg dietary Pueraria extracts to the Wuzhishan piglets’ diets improved the growth performance, immune function, and immune-related gene expression. This study laid a theoretical foundation for popularizing and applying Pueraria extracts in local pig production.

## 1 Introduction

Plant extracts, recognized for their natural and safe properties, have increasingly become a valuable feed additive in livestock and poultry breeding, driven by their wide-ranging benefits (1–3). These benefits extend across multiple dimensions of animal production, such as enhancing growth performance, boosting immune function, improving carcass traits, and elevating meat quality (2, 3). In piglet nutrition, plant extracts have garnered particular attention in recent years. Research has shown that specific plant extracts can optimize piglets’ growth performance and overall health by modulating gut microbiota and enhancing immune responses (4, 5). Essential oils derived from plants like oregano and thyme have demonstrated antimicrobial properties, which can effectively reduce the incidence of gastrointestinal infections in piglets, potentially diminishing the reliance on antibiotics (6). Additionally, Galanopoulos *et al.* explored the antioxidant and anti-inflammatory effects of plant extracts, such as flaxseed oil, which can improve systemic and gut immunity in piglets (7). These findings underscore the necessity of further investigating the applications of plant extracts in piglet nutrition to foster sustainable and healthy livestock production practices. Pueraria extracts are derived from the root of the *Pueraria lobata* plant, native to parts of Asia, including China, Japan, and Korea (8–10). Pueraria extracts contain phytoestrogens, particularly isoflavones such as daidzein and genistein, which have been used in traditional Chinese medicine for centuries due to their various health benefits (9, 11, 12). Wang et al. (13) found that Puerarin improves the immune response, antioxidant capacity, and intestinal morphology of pigeons. Liu et al. (14) reported that the Pueraria crude extracts significantly changed the fecal microbiota of finishing pigs. Guo et al. (15) found that the Pueraria extracts enhanced the antioxidant status and intestinal integrity of broilers. Zhang et al. (16) studied the *Pueraria lobata* extracts, which remediated intestinal dysbiosis and promoted bile acid biosynthesis. Pueraria extracts have been widely utilized in the production of various animals. However, there are relatively few reports regarding their application in piglets, particularly in local pig breeds.

The Wuzhishan pig is a diminutive swine species endemic to China, originating specifically from Wuzhishan in Hainan Province (17). Characterized by its small stature and robust and compact physique, this pig has a relatively small and slightly elongated head, small and thin ears, and an irregular black and white fur distribution. Most individuals exhibit a black back and a white belly, earning them the colloquial name mouse pig (18, 19). The Wuzhishan pig is highly inbred, rendering it an invaluable genetic resource for elucidating the genetic underpinnings of various traits. Owing to its anatomical, physiological, and pathological similarities to humans, it is an ideal biomedical research model. Moreover, the Wuzhishan pig is a crucial breed for the development of high-quality pork products. Its unique characteristics and potential applications underscore the practical significance of studying this pig breed.

The primary aim of this study is to systematically investigate the multifaceted impacts of Pueraria extracts on Wuzhishan piglets. Specifically, we hypothesize that Pueraria extracts can significantly enhance growth performance metrics, such as weight gain and feed efficiency. Additionally, we posit that these extracts can bolster immune function by upregulating key immune-related genes and improving biochemical blood indicators. To achieve these goals, the study will (a) meticulously assess growth parameters and immune responses in piglets administered varying doses of Pueraria extracts; and (b) evaluate the practical applicability of Pueraria extracts in piglet production settings through pilot trials. By focusing on these targeted objectives, the study aims to provide robust scientific evidence supporting the integration of Pueraria extracts into pig production practices, thereby laying a solid theoretical foundation for their widespread adoption.

## 2 Materials and methods

### 2.1 Experiment material

Forty-eight Wuzhishan piglets (sows or castrated boars) with an average body weight of 10.00 ± 1.00 kg, the same genetic background, and similar birth dates were used.

Pueraria extracts (PE) were purchased from Shaanxi Baichuan Biotechnology Co., LTD., Xian, China. The active ingredient contents were 4.37% puerarin, 1.62% daidzin, 1.05% puerarin apigenin, and 0.27% genistin, detected in Liquid chromatography–tandem mass spectrometry.

Serum biochemical kits were purchased from Shanghai Sangon Biotechnology Co., Ltd., Shanghai, China.

### 2.2 Experiment design and sample collection

The amount of Pueraria extracts added was a single influencing factor in this study. Forty-eight Wuzhishan piglets were randomly allocated into four groups, with six replicates and two piglets per replicate. The basal experimental diets for piglets were corn-soybean meal-type diets formulated according to the NCR (2012) standards, and the composition of the basal experimental diets and nutritional levels are shown in Table 1. The control group (Con) was fed basal experimental diets, and the experimental groups were fed 400, 600, and 800 mg/kg Pueraria extracts in powder (PE1, PE2, and PE3, respectively) according to recommended usage. The experiment period was 90 days. The piglet house was maintained at a temperature of 28–30°C and 60–70% humidity. Lighting was provided for 16 h daily. Fecal waste was cleared twice daily, and the house was disinfected weekly with a 0.10% povidone-iodine solution to ensure hygiene.

**Table 1:** Composition (kg/100 kg) of the basal experimental diets^1^ for Wuzhishan piglets.

Ingredients	Contents	Nutrient levels, on an air-dry basis	Contents
Corn, %	32.80	Digestible energy^3^, DE, MJ/kg	13.61
Soybean meal, %	15.40	Crude protein^4^, CP, %	18.00
Wheat, %	8.00	Calcium^4^, Ca, %	0.95
Low protein whey powder, %	6.00	Total Phosphorus^4^, P, %	0.75
Fish meal, %	3.00	Available phosphorus^4^, AP, %	0.53
Corn gluten meal, %	10.00	Lysine^4^, Lys, %	1.25
Wheat bran, %	3.00	Methionine^4^, Met, %	0.35
Soybean oil, %	5.00	Threonine^4^, Thr, %	0.78
Mountain flour, %	0.80	Tryptophan^4^, Try, %	0.29
Calcium hydrogen phosphate, %	2.00		
Sodium chloride, %	0.30		
*L*-Lysine hydrochloride, %	0.60		
Threonine, Thr, %	0.10		
Tryptophan, Try, %	0.10		
Saccharose, %	2.50		
Glucose, %	2.50		
Premix^2^, %	2.00		
Feed mold inhibitor, %	1.00		
Sweetening agent, %	0.30		
Zeolite powder, %	4.60		
Total, %	100.00		

^1^Based on the NRC (2012) nutrient requirements for piglets.

^2^The premix provided the following per kg of diet: VA 2,000 IU, VD 200 IU, VE 45.00 IU, VK 0.50 mg, VB_1_ 1.00 mg, pantothenic acid 12.00 mg, nicotinic acid 10.25 mg, VB_6_ 3.85 mg, VB_12_ 15.00 ug, folic acid 1.35 mg, biotin 0.21 mg, VC 200 mg, Mn (Manganese sulfate) 20.00 mg, Fe (Ferrous sulfate) 80.00 mg, Cu (Copper sulfate) 5.00 mg, I (Potassium iodide) 0.14 mg, Se (Sodium selenite) 0.15 mg.

^3^Calculated value (NRC, 2012).

^4^Analysed content.

At the end of the experiment, all piglets were given 12 h of forbidden feeding and were allowed to drink freely. We chose one piglet that was close to the average weight for slaughter from each replicate. We put one piglet that will be euthanized into a carbon dioxide euthanasia chamber (LC-800-S1, 80 × 70 × 60 cm^3^, Shanghai, China). Once it is calm, the air in the chamber will be replaced with a mixture of 90% carbon dioxide and 10% air at a rate of 15% of the chamber’s volume per minute until the piglet is in a deep state of unconsciousness. The entire process is expected to take about 45 min. Before slaughter, five mL of blood was collected from each pig’s ear vein and centrifuged at 3000 r/min for 15 min. The super serum was collected and divided into 1.50 mL Ep tubes and stored at −20°C for later use. Two grams of jejunum contents were taken from each piglet in 2.00 mL frozen storage tubes and stored in a − 80°C refrigerator for future use. Another section of the jejunum was taken and cut lengthwise to expose the intestinal cavity. Sterile saline was used to clean the intestinal cavity and gently remove the surface intestinal contents. A sterile slide was used to scrape the intestinal mucosa gently, and this process should avoid penetrating the basement membrane. The intestinal mucosa was stored in 2.00 mL frozen storage tubes and placed in a − 80°C refrigerator for later use.

### 2.3 Growth performance

The feed intake of piglets was carefully recorded. The amount of feed offered and the amount wasted by the piglets in each replicate were precisely measured and recorded daily. The average daily feed intake (ADFI) was calculated by subtracting the weight of leftover feed from the initial feed provided. Additionally, each pig was weighed at the end of the experiment to determine the average daily gain (ADG). ADFI, g/d = Total feed intake, g/ Experiment period, d. ADG, g/d = (Final weight, kg - Initial weight, kg) * 1,000/Experiment period, d. Feed conversion ratio (FCR) = ADFI, g/d/ADG, g/d.

### 2.4 Immune function

The immunoglobulin A (IgA), immunoglobulin G (IgG), immunoglobulin M (IgM), complement 3 (C3), complement 4 (C4), total protein (TP), albumin (ALB), alkaline phosphatase (ALP), alanine transaminase (ALT), glutamic oxalacetic transaminase (GOT), and urea nitrogen (UN) in the serum were determined using corresponding commercial assay kits, Shanghai Sangon Biotechnology Co., Ltd., Shanghai, China. The determining process is strictly according to the kit instructions.

### 2.5 Jejunal microorganism

In a sterile super-clean workbench, two grams of jejunum contents were taken and diluted to 10^−7^ with sterile normal saline. Then the total bacteria, *Escherichia coli*, Bacillus acidi lactici, Salmonella, and Saccharomycetes were identified and cultured with LB culture, Macconkey AGAR culture, MRS culture, SS AGAR culture with brilliant green, and Potato glucose AGAR with antibiotics, respectively. The number of various microorganisms was counted using the plate counting method.

### 2.6 RNA extraction and quantitative analysis of mRNA with real-time PCR

Jejunum mucosa samples of soybean size were taken, and RNA was extracted according to the instructions of the animal tissue RNA extraction kit. The quality of RNA was assessed by a 2% agarose gel electrophoresis. An ultra-microspectrophotometer (NanoPhotometer, Implen German) was used to evaluate the total RNA concentration and purity (A260/A280 ratio). The primer sequences are shown in Table 2, and the primers corresponding to the sow gene sequence were synthesized using Sangon (Shanghai, China). The cDNA was synthesized by PrimeScript® RT regent Kit With gDNA Eraser (TaKaRa, Dalian, China) reverse transcription kit. DNA was removed from the sample using a reaction system as 2 μL 5 × gDNA Eraser Buffer, 1 μL gDNA Eraser, 1 μg RNA, then replenished the volume with RNase Free ddH_2_O to 10 μL (20). The system of reverse transcription was 10 μL reaction liquid, 1 μL RT Primer Mix, 4 μL RNase Free ddH_2_O, 4 μL 5 × PrimeScript® Buffer 2, PrimeScript® RT Enzyme MixI. The reaction procedure was 37°C constant temperature for 15 min, 85°C constant temperature for 5 s; 4°C store briefly. The obtained cDNA was stored at −20°C (17). The housekeeping gene of *Glyceraldehyde-3-phosphate dehydrogenase* (*GAPDH*) was used and the relative mRNA levels of *Interleukin-10* (*IL-10*), *Tumor necrosis factor-α* (*TNF-α*), *Mucoprotein 2* (*MUC2*), *Transforming growth factor-β1* (*TGF-β1*), *Zonula occludens-1* (*ZO1*), *Proliferating Cell Nuclear Antigen* (*PCNA*) were calculated using the 2^-ΔΔCt^ method.

**Table 2:** Primer sequence list.

Gene	Gene name	Forward and reverse primers	Product size	Accession No.
*IL-10*	Interleukin-10	F:5’-GTGGCAGCCAGCATTAAGTC-3′	100	NM_214041.1
		R:5’-AACTCTTCACTGGGCCGAAG-3’		
*TNF-α*	Tumor necrosis factor-α	F: 5’-CCAGACCAAGGTCAACCTCC-3’	89	NM_214022.10
		R: 5’-TCCCAGGTAGATGGGTTCGT-3’		
*MUC2*	Mucoprotein 2	F: 5’-CTGTGCGACTACAACTTCGC-3’	142	XM_021082584.1
		R: 5’-AGATGGTGTCGTCCTTGACC-3’		
*TGF-β1*	Transforming growth factor-β1	F: 5’-GGACCTTATCCTGAATGCCTT-3’	134	XM_021093503.1
		R: 5’-TAGGTTACCACTGAGCCACAAT-3’		
*ZO1*	Zonula occludens-1	F: 5’-CTGAGGGAATTGGGCAGGAA-3’	178	XM_021098896.1
		R: 5’-TCACCAAAGGACTCAGCACG-3’		
*PCNA*	Proliferating Cell Nuclear Antigen	F: 5’-GTGATTCCACCACCATGTTC-3’	123	NM_001291925.1
		R: 5’-TGAGACGAGTCCATGCTCTG-3’		
*GAPDH*	Glyceraldehyde-3-phosphate dehydrogenase	F: 5’-GTCGGAGTGAACGGATTTGG-3’	76	NM_001206359.1
		R: 5’-CAATGTCCACTTTGCCAGAGTTAA-3’		

### 2.7 Statistical analysis

Statistical analyses were conducted using SPSS 29.0 statistics software (NY, USA). The sample size for this study was determined based on the anticipated effect size, statistical power, and significance level. Drawing on prior research and pilot data, we set the expected effect size at 0.50, the significance level at 0.05, and the statistical power at 0.80. Utilizing G*Power software for the calculation, we determined that a minimum of six piglets per group was required. Accordingly, the study was structured with six replicates per group, each comprising two piglets, for a total of 48 piglets. This design ensures the robustness of our findings and achieves the desired statistical power. The Kolmogorov–Smirnov test was used to check if all data in this study followed a normal distribution. Data were expressed as mean ± SEM. Statistical comparisons between treatments were conducted using one-way ANOVA or Welch ANOVA following Kolmogorov–Smirnov and variance homogeneity tests (Barteet’s or Levene’s test). To mitigate the risk of Type I errors due to multiple comparisons, we employed the Bonferroni correction method to adjust the *p*-values. The test results of all analyses were considered significant at *p* < 0.05.

## 3 Results

### 3.1 Effects of Pueraria extracts on the growth performance of Wuzhishan piglets

There were no significant differences (*p* > 0.05) in initial weight and ADFI among groups. The final weight and ADG were higher (*p* < 0.05), and the FCR was lower (*p* < 0.05) in PE2 and PE3 compared with the Con. (Table 3).

**Table 3:** Effects of Pueraria extracts on the growth performance of Wuzhishan piglets.

Items	Con	PE1	PE2	PE3	*P*-value
Initial weight, kg	10.00 ± 1.01	10.00 ± 0.98	10.00 ± 0.96	10.00 ± 1.02	0.1206
Final weight, kg	24.62 ± 0.35^b^	25.12 ± 0.46^b^	26.56 ± 0.16^a^	26.51 ± 0.22^a^	0.0352
ADFI, g/d	583 ± 10.26	580 ± 10.26	570 ± 6.92	572 ± 8.12	0.2315
ADG, g/d	162 ± 5.76^b^	168 ± 3.05^b^	184 ± 5.06^a^	183 ± 6.23^a^	0.0238
FCR	3.60 ± 0.12^a^	3.45 ± 0.10^a^	3.10 ± 0.10^b^	3.12 ± 0.11^b^	0.0389

^a,b^Values with different small letter superscripts in the same column mean a significant difference (*p* < 0.05). ADFI: average daily feed intake; ADG: the average daily gain; FCR: feed conversion ratio.

### 3.2 Effects of Pueraria extracts on serum immune indices of Wuzhishan piglets

There was no significant difference (*p* > 0.05) in lgA contents among groups. The lgG contents in PE3 were higher (*p* < 0.05) compared with the Con. The lgM and C3 contents in PE2 and PE3 were higher (*p* < 0.05) than in the Con. The C4 contents were higher (*p* < 0.05) among groups treated with Pueraria extracts compared with the Con. (Table 4).

**Table 4:** Effects of Pueraria extracts on serum immune indice of Wuzhishan piglets.

Items	Con	PE1	PE2	PE3	*P*-value
IgA, g·L^−1^	0.03 ± 0.01	0.04 ± 0.01	0.04 ± 0.02	0.03 ± 0.02	0.0968
IgG, g·L^−1^	3.12 ± 0.21^c^	3.56 ± 0.29^b^	3.97 ± 0.12^ab^	4.13 ± 0.17^a^	0.0452
IgM, g·L^−1^	0.88 ± 0.07^b^	0.90 ± 0.03^b^	1.12 ± 0.01^a^	1.11 ± 0.02^a^	0.0311
C3, mg·L^−1^	30.52 ± 1.56^b^	31.99 ± 2.01^b^	36.25 ± 1.26^a^	36.86 ± 1.37^a^	0.0205
C4, mg·L^−1^	26.59 ± 1.23^b^	31.26 ± 1.56^a^	32.23 ± 1.75^a^	32.46 ± 2.01^a^	0.0289

^a,b,c^Values with different small letter superscripts in the same column mean a significant difference (*p* < 0.05). IgA, immunoglobulin A; IgG, immunoglobulin G; IgM, immunoglobulin M; C3, complement 3; C4, complement 4.

### 3.3 Effects of Pueraria extracts on serum biochemical indices of Wuzhishan piglets

The serum TP and UN contents among groups were not significantly different (*p* > 0.05). The serum ALB and ALP contents in groups treated with the Pueraria extracts were higher (*p* < 0.05) compared with the Con. The ALT and GOT contents were lower (*p* < 0.05) in groups treated with Pueraria extracts than in the Con. (Table 5).

**Table 5:** Effects of Pueraria extracts on serum biochemical indices of Wuzhishan piglets.

Items	Con	PE1	PE2	PE3	*P*-value
TP, g·L^−1^	80.17 ± 16.03	83.23 ± 8.95	81.19 ± 9.12	83.05 ± 10.12	0.0859
ALB, g·L^−1^	36.03 ± 2.12^b^	39.29 ± 1.06^a^	39.72 ± 1.07^a^	39.00 ± 2.61^a^	0.0416
ALP, U·L^−1^	230 ± 5.12^c^	270 ± 10.56^b^	262 ± 10.39^b^	290 ± 6.31^a^	0.0357
ALT, U·L^−1^	99.02 ± 5.87^a^	80.12 ± 6.95^b^	76.25 ± 6.12^bc^	67.19 ± 4.12^c^	0.0267
GOT, U·L^−1^	190 ± 10.12^a^	156 ± 10.92^b^	120 ± 10.11^c^	117 ± 9.32^c^	0.0379
UN, mmol·L^−1^	5.87 ± 0.42	5.32 ± 1.25	5.27 ± 2.17	5.00 ± 2.29	0.9150

^a,b,c^Values with different small letter superscripts in the same column mean a significant difference (*P* < 0.05). TP, total protein; ALB, albumin; ALP, alkaline phosphatase; ALT, alanine transaminase; GOT, glutamic oxalacetic transaminase; UN, urea nitrogen.

### 3.4 Effects of Pueraria extracts on the jejunum flora of Wuzhishan piglets

There was no significant difference (*p* > 0.05) in total bacteria contents among groups. The *Escherichia coli* and Salmonella contents among groups treated with Pueraria extracts were lower (*p* < 0.05) compared with the Con. The Saccharomycetes and Bacillus acidi lactici contents among groups treated with Pueraria extracts were higher (*p* < 0.05) than in the Con. (Table 6).

**Table 6:** Effects of Pueraria extracts on jejunum flora of Wuzhishan piglets.

Items	Con	PE1	PE2	PE3	*P*-value
Total bacteria, lgCFU·g^−1^	11.62 ± 0.18	11.65 ± 0.12	11.62 ± 0.16	11.63 ± 0.25	0.0951
*Escherichia coli*, lgCFU·g^−1^	6.59 ± 0.25^a^	5.56 ± 0.26^b^	5.00 ± 1.13^c^	5.02 ± 1.09^c^	0.0326
Bacillus acidi lactici, lgCFU·g^−1^	6.24 ± 0.16^c^	7.15 ± 0.19^b^	7.20 ± 0.20^b^	7.56 ± 0.18^a^	0.0215
Salmonella, lgCFU·g^−1^	5.12 ± 0.08^a^	4.67 ± 0.10^b^	4.32 ± 0.09^c^	4.33 ± 0.01^c^	0.0456
Saccharomycetes, lgCFU·g^−1^	5.00 ± 0.16^c^	5.36 ± 0.10^b^	5.69 ± 0.12^a^	5.72 ± 0.14^a^	0.0411

^a,b,c^Values with different small letter superscripts in the same column mean a significant difference (*P* < 0.05).

### 3.5 Effects of Pueraria extracts on expression of immune-related genes in Wuzhishan piglets

**Figure 1:**
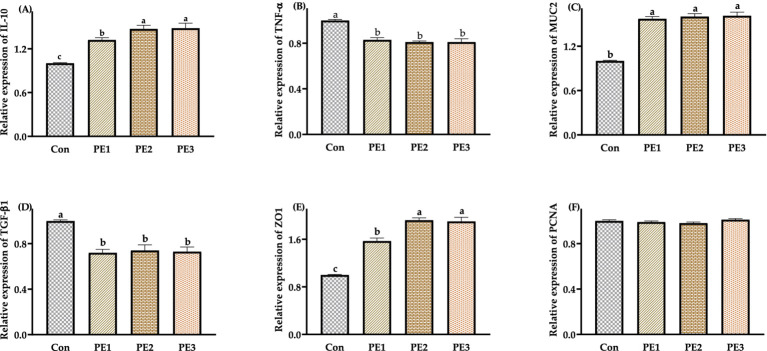
Effects of Pueraria extract on the expression of immune-related genes in jejunum mucosa of Wuzhishan piglets. **(A)** The data of relative expression levels of *IL-10* (*Interleukin-10*) in Con, PE1, PE2, and PE3 were 1.00 ± 0.01^c^, 1.32 ± 0.03^b^, 1.47 ± 0.05^a^, 1.48 ± 0.07^a^, individually, *p* = 0.0237; **(B)** The data of relative expression levels of *TNF-α* (*Tumor necrosis factor-α*) in Con, PE1, PE2, and PE3 were 1.00 ± 0.01^a^, 0.83 ± 0.02^b^, 0.81 ± 0.01^b^, 0.81 ± 0.02^b^, individually, *p* = 0.0429; **(C)** The data of relative expression levels of *MUC2* (*Mucoprotein 2*) in Con, PE1, PE2, and PE3 were 1.00 ± 0.01^b^, 1.57 ± 0.03^a^, 1.60 ± 0.04^a^, 1.61 ± 0.05^a^, individually, *p* = 0.0436; **(D)** The data of relative expression levels of *TGF-β1* (*Transforming growth factor-β1*) in Con, PE1, PE2, and PE3 were 1.00 ± 0.01^a^, 0.97 ± 0.04^b^, 0.72 ± 0.03^c^, 0.74 ± 0.05^c^, individually, *p* = 0.0268; **(E)** The data of relative expression levels of *ZO1* (*Zonula occludens-1*) in Con, PE1, PE2, and PE3 were 1.00 ± 0.01^c^, 1.57 ± 0.05^b^, 1.92 ± 0.04^a^, 1.90 ± 0.07^a^, individually, *p* = 0.0357; **(F)** The data of relative expression levels of PCNA (*Proliferating Cell Nuclear Antigen*) in Con, PE1, PE2, and PE3 were 1.00 ± 0.01, 0.99 ± 0.01, 0.98 ± 0.01, 1.01 ± 0.01, individually, *p* = 0.0689. ^a,b,c^Values with different small letter superscripts in the same column mean a significant difference (*p* < 0.05).

The relative expressions of *IL-10*, *ZO1,* and *MUC2* in jejunum mucosa in groups treated with Pueraria extracts were higher (*p* < 0.05) than those in the Con, and the relative expressions of *IL-10* and *ZO1* in jejunum mucosa were higher (*p* < 0.05) in PE2 and PE3 compared with PE1. The relative expressions of *TNF-α* and *TGF-β1* in jejunum mucosa were lower (*p* < 0.05) in PE1, PE2, and PE3 compared with the Con. There was no significant difference (*p* > 0.05) in the relative expressions of *PCNA* in jejunum mucosa among groups (Figure 1).

## 4 Discussion

Growth performance is a key indicator of animal growth, which is significant for evaluating animal growth environment and feed nutrition (21–23). The isoflavones in Pueraria extract, such as puerarin, daidzein, and genistein, have essential medicinal value; they have anti-inflammatory, antioxidant, anti-tumor, and improve immunity (24, 25). Guo et al. (15) found that the Pueraria extract supplementation decreased the average daily gain during the whole period and improved the feed conversion ratio of broiler chickens. Gong et al. (26) added the *Pueraria lobata* leaf powder to the feed of broiler chickens and found it did not affect the growth and slaughter performance. Wang et al. (13) found that the puerarin treatment did not significantly alter the growth performance of pigeons. Tiyasatkulkovit et al. (27) studied the effect of Pueraria Mirifica extract and puerarin on the healthy growth of primary baboon osteoblasts. In this study, the Pueraria extract improved the final body weight, the average daily gain, and the feed conversion ratio of Wuzhishan piglets, possibly because Pueraria extract affects mammals more than birds. At the same time, the isoflavones in Pueraria extracts may improve the growth performance of pigs through multiple mechanisms, such as regulating metabolic pathways and hormone levels, improving gut health, and exerting antioxidant and anti-inflammatory effects.

The animal immune system is a set of complex biological mechanisms that enable the animal to recognize and defend against foreign pathogens, such as bacteria, viruses, parasites, etc., while also removing damaged or aging cells in the body and maintaining the stability of the internal environment (28–30). Plant extracts have received attention for their naturalness, versatility, and potential animal health and immunity benefits. They contain various bioactive substances, such as alkaloids, polyphenols, flavonoids, polysaccharides, and volatile oils, which are closely related to immune function (2, 3). Immunoglobulins are a class of globulins with antibody activity or molecular chemical structure similar to antibodies, and they play an essential role in the animal immune system. B lymphocytes secrete immunoglobulin after being stimulated by an antigen, mainly in serum, body fluid, exocrine fluid, and some cell membranes, and have the efficacy and effect of anti-infection, improving immunity, and preventing and treating diseases (31–33). The complement C3 and C4 in serum are critical immune components that play a key role in animal immune response and inflammation. Complement C3 is the most abundant complement component in serum, while C4 is involved in the classical activation pathway of complement, and their content can reflect inflammation and immune system problems (34–36). Gong et al. (26) found that *Pueraria lobata* leaf powder improved the thymus organ index, antioxidant performance, and immune functions of broiler chickens. Wang et al. (13) found that the puerarin treatment afforded a significant linear enhancement in the thymus index and significantly increased serum immunoglobulin A and immunoglobulin M levels in pigeons in a linear manner of pigeons. Jung et al. (37) studied the effects of *Lactobacillus casei* HY2782 and *Pueraria lobata* root extract complex, and they found that it significantly reduced Th2/Th17-derived cytokines, immunoglobulin E, and leukotriene C4 in bronchoalveolar lavage fluid and serum in mice. In this study, the Pueraria extract improved the serum immunoglobulins (G and M) and complements (C3 and C4) of Wuzhishan piglets, which suggests that Pueraria extract can improve the immune performance of piglets by increasing the immunoglobulins and complements in the body.

Albumin, alkaline phosphatase, alanine aminotransferase, and glutamic oxalate aminotransferase are all critical indicators of animal immune function (38–40). Albumin is the most abundant protein in plasma, which can combine with many small insoluble molecules of organic matter and inorganic ions in the body to form soluble complexes and become the transport form of these substances in blood circulation (38). Albumin levels can reflect the nutritional status and immune function in animals, and low levels may indicate malnutrition or impaired immune function (39). Alkaline phosphatase is a nonspecific phosphohydrolase that can catalyze the hydrolysis of monophosphate and transfer phosphate groups (40). Alanine aminotransferase and glutamic oxalate aminotransferase are commonly used to assess liver health because they are highly active in liver cells (40). When liver cells are damaged, these enzymes are released into the blood, resulting in elevated serum aminotransferase activity (39, 40). In this study, the Pueraria extracts increased the albumin and alkaline phosphatase contents, and they decreased alanine aminotransferase and glutamic oxalate aminotransferase contents, which suggests that Pueraria extract has the function of protecting the animal liver, thereby directly or indirectly improving animal immune function.

*Escherichia coli*, Lactobacillus, Salmonella, and Saccharomycetes affect the balance of the gut microbiota through interactions with host cells, which impact the immune system (41–44). Probiotics such as Lactobacillus and Saccharomycetes usually help to enhance the immune response (45). In contrast, pathogens such as Salmonella and pathogenic *Escherichia coli* may activate the immune response to clear the infection (46, 47). Kwon et al. (48) found that *Pueraria lobata* extract increased the *Lactobacillus rhamnosus* in human fecal specimens. Xu et al. (49) found that the polyphenols extracted from *Pueraria lobata* root improved the gut microbiota in mice, and Chen et al. (50) reported that the polysaccharides from *Pueraria lobata* significantly increased the abundance of beneficial bacteria, including Oscillospira and Anaerotruncus. In this study, Pueraria extracts significantly increased the Saccharomycetes content while decreasing the content of *Escherichia coli* and Salmonella in the jejunum of Wuzhishan piglets. This finding directly demonstrates that Pueraria extracts have the ability to inhibit the growth of harmful bacteria while simultaneously promoting the growth of beneficial bacteria. These changes may indirectly enhance the immune function of pigs by improving gut barrier function and reducing the risk of pathogen infection, thereby improving the immunity of piglets, which is consistent with the improved immune indicators described above.

Interleukin-10 (IL-10) is a pleiotropic cytokine with immunosuppressive or immunostimulating effects (51). Mucin 2 (MUC2) is a significant component of the intestinal mucus layer, which has the functions of lubricating the intestinal tract, providing antimicrobial proteins and adhesion sites for commensal flora, and resisting the invasion of pathogenic bacteria and harmful substances (52). Zonula occludens 1 (ZO1) is a tight junction protein that plays a vital role in forming and maintaining tight junctions between cells (53). Tumor necrosis factor-*α* (TNF-α) is an important cytokine that regulates inflammation, apoptosis, and immune cell activity (54). Transforming growth factor-β1 (TGF-β1) is a multifunctional cytokine synthesized by almost all cells and has the functions of immune regulation and promoting fibrosis (55). IL-10, MUC2, ZO1, TNF-*α*, and TGF-β1 play essential roles in the animal immune system and intestinal health, and they are involved in regulating the immune response and maintaining the integrity of the intestinal barrier through different mechanisms. In this study, the relative expressions of *IL-10*, *MUC2,* and *ZO1* were increased, and the relative expressions of *TNF-α* and *TGF-β1* were decreased in the groups treated with Pueraria extracts, which may be because the changes in the microbial community described above, by improving gut barrier function and reducing the risk of pathogen infection, indirectly affected the function of the immune system. It can be speculated that the alterations in immune-related gene expression are likely associated with the changes in the gut microbial community, which will be further explored in future studies.

The Wuzhishan pig, a local pig breed, is distinguished by its small size and relatively slow growth rate, resulting in a longer growth cycle compared to other pig breeds. Typically, it requires a longer period to reach market weight. Although the study period allowed for the observation of significant improvements in growth performance and immune function of Wuzhishan pigs due to Pueraria extracts, it may not fully reflect the potential long-term impacts of prolonged use of Pueraria extracts on piglets. In this study, Pueraria extracts significantly enhanced the average daily gain and feed conversion ratio of Wuzhishan pigs. In the long term, these improvements may help piglets maintain better growth efficiency throughout their entire growth cycle, thereby reducing feeding costs. Pueraria extracts also increased the immunoglobulins and complement levels in piglets within a short period, suggesting that they may have a positive impact on the long-term immune function of piglets. It can be inferred that long-term use of Pueraria extracts may enhance the immune system of piglets, reduce disease incidence, and consequently improve the overall health status of the pig herd. Moreover, Pueraria extracts improved the number of beneficial bacteria in the piglets’ intestines while reducing the number of harmful bacteria in the short term. These changes may further optimize the gut microbiota structure and enhance gut barrier function over the long term, thereby reducing the incidence of intestinal diseases. However, while Pueraria extracts have shown positive effects on growth and immunity in the short term, long-term use may alter the piglets’ requirements and absorption of other nutrients. This could lead to the development of tolerance to Pueraria extracts, thereby reducing their effectiveness in later stages. Therefore, further research is needed to fully understand the long-term effects and safety of Pueraria extracts in piglet diets.

## 5 Conclusion

In this study, adding dietary Pueraria extracts at 600 mg/kg to the diets of Wuzhishan piglets significantly improved growth performance by increasing final weight and average daily gain, while decreasing the feed conversion ratio. These findings indicate that Pueraria extracts can enhance feed efficiency and promote piglet growth, potentially leading to economic benefits for pig farming. Additionally, Pueraria extracts enhanced immune function by boosting the levels of immunoglobulin G and M and complement 3 and 4, and by promoting beneficial bacteria such as Lactobacillus and Saccharomycetes in the jejunum. These results suggest that Pueraria extracts can be used as a natural and safe feed additive to improve the health status of piglets, thereby reducing the risk of diseases and the need for antibiotics. Future research endeavors will be dedicated to unraveling the intricate mechanisms through which Pueraria extracts exert their beneficial effects. This will involve investigating their role in regulating metabolic pathways and modulating the gut microbiota.

## Data Availability

The raw data supporting the conclusions of this article will be made available by the authors, without undue reservation.
